# Spectral Weighting of Monaural Cues for Auditory Localization in Sagittal Planes

**DOI:** 10.1177/23312165251317027

**Published:** 2025-03-18

**Authors:** Pedro Lladó, Piotr Majdak, Roberto Barumerli, Robert Baumgartner

**Affiliations:** 1 Acoustics Lab, Department of Information and Communication Engineering, Aalto University, Espoo, Finland; 2 Institute of Sound Recording, Department of Music and Media, 3660 University of Surrey, Guildford, UK; 3 Acoustics Research Institute, 31390 Austrian Academy of Sciences, Vienna, Austria

**Keywords:** sound localization, auditory model, sagittal-plane localization, monaural spectral cues, spectral weighting

## Abstract

Localization of sound sources in sagittal planes significantly relies on monaural spectral cues. These cues are primarily derived from the direction-specific filtering of the pinnae. The contribution of specific frequency regions to the cue evaluation has not been fully clarified. To this end, we analyzed how different spectral weighting schemes contribute to the explanatory power of a sagittal-plane localization model in response to wideband, flat-spectrum stimuli. Each weighting scheme emphasized the contribution of spectral cues within well-defined frequency bands, enabling us to assess their impact on the predictions of individual patterns of localization responses. By means of Bayesian model selection, we compared five model variants representing various spectral weights. Our results indicate a preference for the weighting schemes emphasizing the contribution of frequencies above 8 kHz, suggesting that, in the auditory system, spectral cue evaluation is upweighted in that frequency region. While various potential explanations are discussed, we conclude that special attention should be put on this high-frequency region in spatial-audio applications aiming at the best localization performance.

## Introduction

Monaural spectral cues allow humans to localize a broadband sound source within a sagittal plane (Middlebrooks, 1999b; Van Wanrooij & Van Opstal, 2004). These cues originate from the direction-dependent filtering of the torso, the head, and mainly the pinnae (Shaw, 1974), which is usually described by the head-related transfer functions (HRTFs) and emphasized at frequencies above 700 Hz (Algazi et al., 2001; Baumgartner et al., 2014). The auditory system is sensitive to these spectral differences due to its tonotopic organization (Von Békésy & Wever, 1960), but the exact mechanisms describing the extraction of spectral cues from this sound representation are still not fully clarified. Neurophysiological measurements of the dorsal cochlear nucleus in cats showed sensitivity to positive spectral gradients (PSGs) of the sound that could be central to sagittal-plane localization (Reiss & Young, 2005). This finding, combined with computational considerations (Zakarauskas & Cynader, 1993), led to the integration of PSG extraction in a sagittal-plane sound-localization model, which markedly improved the quality of the prediction of human behavioral data (Baumgartner et al., 2014). The PSG profiles emphasize local features such as spectral peaks and notches, while reducing the influence of macroscopic spectral characteristics such as spectral tilt (Macpherson & Sabin, 2013). However, further research is needed to understand the relative contribution of monaural cues on sound source localization.

The relative contribution of monaural cues in various frequency regions may be estimated from the acoustic filtering of the pinna. This pinna filtering provides crucial spatial information because it varies with the polar angle, θ ∈ [−90°, 270°), from front below to rear below within a sagittal plane, as defined by Macpherson and Middlebrooks (2000). Zonooz et al. (2019) studied the contribution of the most prominent spectral notch in the HRTF for elevation, since its center frequency tends to present a monotonic increase over this dimension. Their results showed that the main notch region (NR) is key for elevation perception, but they found neighboring frequency regions to also play a role. Other acoustic factors, such as secondary notches in the HRTF, seem to be crucial for localization in sagittal planes (Macpherson & Sabin, 2013; Middlebrooks & Green, 1991). Thus, it is not surprising that the ability of the listeners to discriminate between front and back drops when the notches in the HRTFs are flattened (Zhang & Hartmann, 2010). The pinnae size is another example of the potential contribution of acoustic factors (Middlebrooks, 1999a). This is evident from the results of a speech localization study investigating the role of high frequencies (Best et al., 2005). For stimuli low-pass filtered at 8 kHz, Best et al. (2005) found interindividual differences that seemed related to the subjects’ ear size. Taken together, the consideration of acoustic factors for understanding the across-frequency integration of spectral cues is crucial, but it seems to not fully explain the individual differences in sagittal-plane localization of sounds.

The contribution of monaural cues has also been studied from a purely perceptual point of view (Langendijk & Bronkhorst, 2002; Zhang & Hartmann, 2010). In a battery of perceptual tests, Zhang & Hartmann (2010) systematically modified the spectral content of stimuli, for example, by flattening certain frequency regions, in a front-back discrimination task (DT). They found that the contribution of each frequency region is extremely individual even in a simple front-back DT, suggesting that listeners might have developed individual localization strategies, the origin of which remained unclear. Ahrens and Brimijoin (2019) conducted a study in which listeners were asked to indicate whether a target stimulus was below or above a reference stimulus, both of which consisted of a combination of simultaneously presented narrowband noises. For reference, all the narrowband noises were filtered by the HRTF corresponding to a single direction. For the targets, each narrowband noise was filtered with the HRTF corresponding to a random direction. The relative contribution of a spectral region to the perceived direction was then computed from the relation between the subject responses and the direction of the targets in each frequency band. The results suggested an emphasized contribution of the frequency region around 6 kHz. However, the large variation across subjects indicates that the contribution of this frequency region was strongly individual.

Understanding which frequency ranges contribute more to the process of sound localization is essential for spatial-audio applications, such as hearing aids or virtual-reality audio systems. In order to shed light on the spectral contribution of spatial cues, we compared the effect of five spectral weighting schemes on the performance of an auditory model at predicting sound localization of wideband, flat-spectrum targets. The sound localization model, originally introduced by Baumgartner et al. (2014) and modified here, aims at predicting the probability distribution of spatial responses of a listener to a target stimulus. We hypothesized that the studied modification of spectral weighting schemes would affect the model-estimated distributions, even for wideband, flat-spectrum stimuli, which are considered easy to localize compared to band-limited or other nonflat-spectrum stimuli. In turn, the attempt to identify listeners’ spectral weighting functions via model fitting assumes that the distribution of listeners’ responses to these easy-to-localize stimuli is sensitive to the weighting function they employ. Accordingly, we hypothesized that there is a spectral weighting scheme that would best predict the observed response distribution of a listener in the actual localization experiment. The results of our comparison might help to determine which frequency ranges need to be accurately delivered to the listener in spatial-audio applications.

## Methods

Five spectral weighting schemes were compared by analyzing their impact on the predictions of sagittal-plane response distributions. The predictions used the sagittal-plane localization model from Baumgartner et al. (2014), modified to accommodate the spectral weighting schemes. Each model variant was reparametrized to guarantee a fair comparison across spectral weighting schemes. The prediction quality of the five model variants was compared to actual localization data from various studies by means of Bayesian statistics. For each model variant, we report the goodness of fit.

### Actual Localization Data and Directional Transfer Functions

We used the localization responses from Goupell et al. (2010); Majdak et al. (2010); Majdak et al. (2013a); and Majdak et al. (2013b). These were obtained in experiments with normal-hearing human listeners localizing virtual sound sources, generated by convolving a 500-ms white-noise burst with the listeners’ individual directional transfer functions (DTFs; i.e., HRTFs with the direction-independent characteristics removed for each ear; Middlebrooks, 1999b). These DTFs are available in the Auditory Modeling Toolbox (AMT; Majdak et al., 2022) as accompanying data for the model implementation for Baumgartner et al. (2014). The localization responses were given using a manual-pointing method, that is, the listener pointed with a pointer in the hand toward the perceived sound direction (Haber et al., 1993; Majdak et al., 2010). In order to reduce a potential misalignment between the actual pointer direction and targeted response direction, a visual cursor presented via a head-mounted display in a virtual environment showed the actual pointing direction during the whole response procedure. With that visual information, any misalignment could have been corrected by the listener before confirming the direction with a click. The responses were collected after procedural training, in which a visual target had to be hit with a root-mean-squared error of 2° measured in three consecutive trials. The procedural training aimed at reducing the misalignment between the perceived and the reported direction. For further methodology details, see the corresponding studies.

The pooled data set comprised 23 humans (14 female, 9 male; between 19 and 46 years; all audiometrically normal hearing) and is summarized in Table 1 by means of two localization metrics. The first metric is the quadrant error rate (QE, in %), which measures the rate of nonlocal responses, that is, with an absolute angular disparity between target and response polar angles (PE) larger than 90°. The second metric is the local PE (in degrees), which captures both the accuracy and the precision of the local responses, that is, with an absolute PE smaller than or equal to 90°, by computing the polar root-mean-square error (Middlebrooks, 1999a). These metrics were also used in Baumgartner et al. (2014) for the same experimental data to parameterize and validate the model and are available in the AMT (Majdak et al., 2022).

**Table 1.** Localization Metrics of the Actual Localization Responses Used in Our Study.

Median Plane				Sagittal Planes			
ID	*N*	QE	PE	ID	*N*	QE	PE
NH43	48	0	36.25	NH58	226	1.33	27.5
NH53	79	1.27	24.83	NH46	250	3.6	32.16
NH58	74	1.35	27.61	NH12	2079	3.94	28.62
NH15	383	1.83	34.48	NH53	221	4.98	32.52
NH42	278	3.24	29.85	NH71	253	6.72	34.95
NH12	721	3.47	30.31	NH15	1717	6.81	33.72
NH46	50	6.0	33.9	NH21	159	6.92	34.08
NH16	414	6.52	30.78	NH14	256	7.81	30.84
NH14	44	9.09	23.96	NH16	1685	8.96	35.93
NH17	150	9.33	36.14	NH42	22	9.09	31.33
NH57	42	9.52	24.61	NH22	113	10.62	32.19
NH68	98	10.2	32.6	NH43	252	10.71	37.15
NH64	141	11.35	33.47	NH39	369	11.65	40.49
NH62	101	11.88	37.05	NH17	650	12.15	35.98
NH22	187	12.3	32.49	NH64	459	12.42	35.6
NH21	141	12.77	34.2	NH68	502	12.75	36.63
NH72	117	12.82	36.55	NH55	257	13.62	41.18
NH39	231	13.42	36.11	NH72	483	13.66	41.29
NH33	149	14.09	37.62	NH57	258	19.77	33.94
NH71	47	14.89	37.18	NH33	151	20.53	33.22
NH55	43	16.28	34.27	NH62	499	21.44	38.18
NH18	136	18.38	38.34	NH41	161	22.36	38.03
NH41	139	19.42	38.33	NH18	614	22.8	39.43

ID: Listeners’ identifier; *N*: Number of trials; QE: Quadrant error rate (in %); PE: Local polar error (in degrees). Median plane comprises data from lateral range: −10° < ϕ < 10°. Lateral sagittal planes comprise all remaining data outside the median plane ( ϕ <−10° ∪ ϕ >10°). Data from Goupell et al. (2010); Majdak et al. (2010); Majdak et al. (2013a); and Majdak et al. (2013b). The listeners are sorted by QE for median plane (left column) and sagittal plane (right column) localization data.

### Sagittal-Plane Localization Model

We based our work on a sagittal-plane sound-localization model (Baumgartner et al., 2014), which infers the probability distribution of the listener's polar angle responses by comparing the spectral cues derived from the target sound to internally stored cue templates. In that model, the cues are derived from the listener's DTFs. To extract the monaural cues of the target and the template, first, the incoming sound is processed by a peripheral auditory model that computes the spectral magnitude profile ξ as the log of the average squared amplitude (in dB), with the average calculated within each band of a Gammatone filterbank (Patterson, 1994). The center frequencies of the Gammatone filters were spaced by one equivalent rectangular bandwidth between 700 Hz and 18 kHz. From this magnitude profile, the PSG profile $\tilde{\xi }$ is computed as:

$$\tilde{\xi }[b]=\max(\xi [b]-\xi [b-1],0),$$

where *b* denotes the frequency band. The PSG profile of the target is then compared to each of the templates by computing a distance metric. In our study, in order to integrate the spectral weighting schemes, we modified the original model by including weights in the calculation of the distance metric:

$$d[\theta ]=\overset{N_{b}}{\underset{b=2}{\sum }}\;|\tilde{\xi_{r}}[\theta ,b]-\tilde{\xi_{t}}[b]|\cdot w[b].$$

where *d* is the distance metric, ${\tilde{\xi }}_{r}$ is the template PSG profile, $\tilde{\xi_{t}}$ is the target PSG profile, and w[b] is the weight assigned to the frequency band *b* (the specific weighting schemes are introduced in the following section *Model Variants: Weighting Schemes*). All subsequent stages of the model from Baumgartner et al. (2014) were not modified. Hence, as the next step, the weighted distance metric was mapped to a similarity index by applying the following sigmoid function (Baumgartner et al., 2014):

$$\varsigma [\theta ]=1-(1+e^{\text{-}\Gamma (d[\theta ]-S)}{)}^{-1},$$

where ς is the similarity index, Γ is the degree of selectivity, which modifies the slope of the psychometric function, and *S* is the listener-specific sensitivity, which shifts the psychometric function. Conceptually, the degree of selectivity represents the ability of the listeners to estimate spectral similarity from gradual to abrupt. The sensitivity represents the ability of the listener to discriminate among internal representations.

As in Baumgartner et al. (2014), ς was calculated for each ear separately. To combine the information from both ears, the binaural weighting of the ears in the process of sagittal-sound localization was considered (Macpherson & Sabin, 2007; Morimoto, 2001). To this end, the binaural similarity index was calculated as the α -weighted sum of the two similarity indices for each ear, with α being larger for the ipsilateral ear (as shown in Fig. 2 of Baumgartner et al., 2014). The binaural similarity index represents the result from the auditory perception process. In order to further model the uncertainties in the pointing process of the subject during the task, that is, after localizing the sound, the sensorimotor scatter ε (the third model parameter) smeared the mapping between the perceived direction and the given response in a random process following a Gaussian distribution. These model responses were then normalized to a probability mass vector (PMV) that describes the probability of observing a response at a given polar angle given the target sound. Note that the PMV describes a probability distribution of responses, not a single response usually recorded in an actual localization task, and the model does not include a decision stage that estimates such a single response. Since the PMV is computed from the entire distance function, it is sensitive to the spectral weighting scheme.

**Figure 1.**
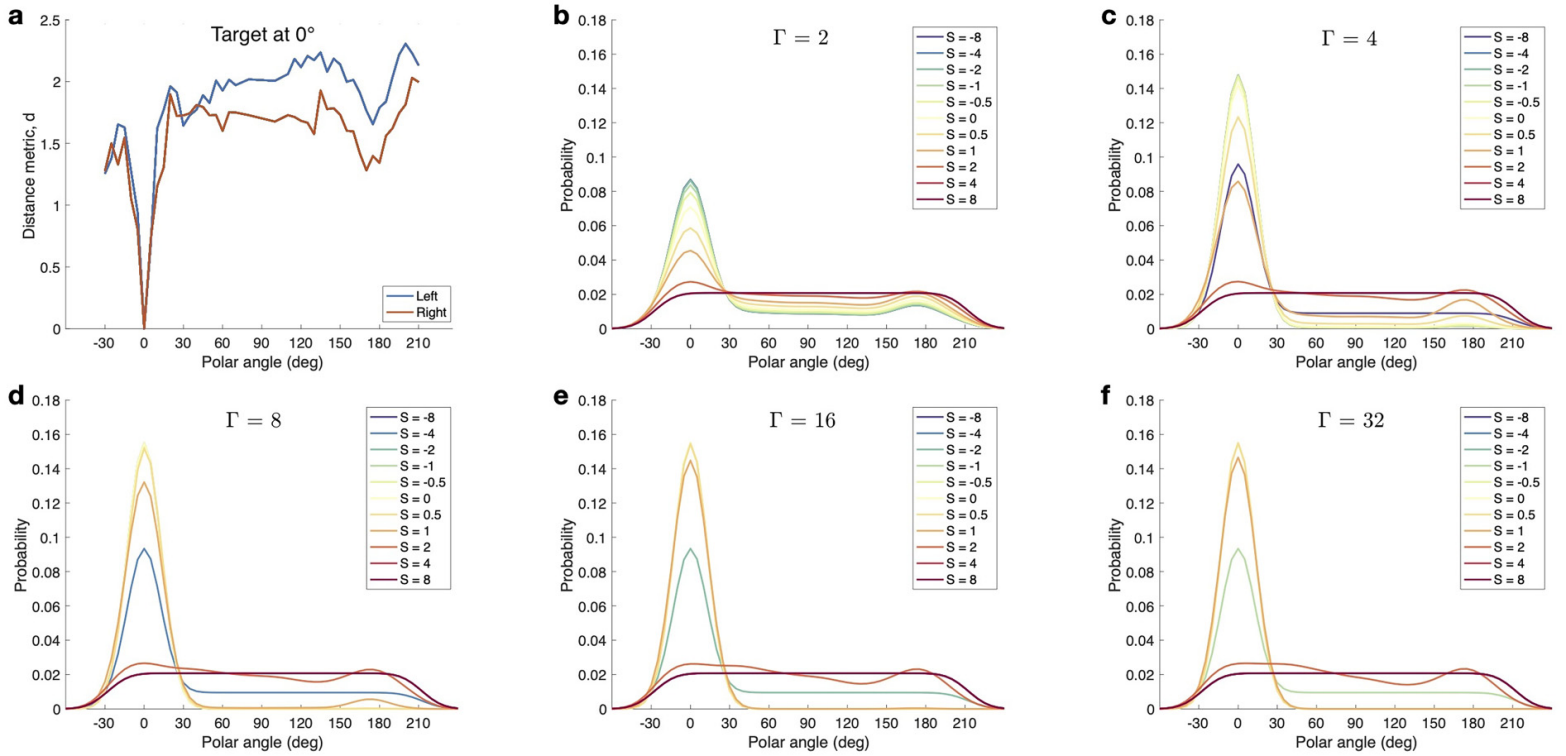
Effect of the parameters Γ and *S* on the probability mass vectors (PMVs). Panel (a) shows the results of computing the distance metric using the Flat scheme for a target located at θ = 0° in the median plane for the example listener NH12. Panels (b) to (f) show the corresponding PMVs obtained for systematically increased parameters Γ and *S*. For this example, the parameter ε = 12.79° is fixed at the group-level average (see Table 2 in Supplemental Material).

To illustrate the effect of the model parameters Γ and *S* on the resulting PMV, Figure 1 shows modeled PMVs obtained for an example listener (NH12) using a range of reasonable parameter values. Panel (a) shows the distance metric for various polar angles along with the median plane and the target located at θ = 0°, for both ears. The panels (b) to (f) show the corresponding PMVs obtained for various values of Γ with *S* as independent parameter. The model parameters mainly affect the PMV by amplifying or compressing the differences in the distance metric. Higher Γ results in sharper PMVs and larger differences across *S* parameters. Very low and high *S* results in shallow PMVs and the *S* leading to the sharpest PMVs depends on Γ.

### Model Variants: Weighting Schemes

**Figure 2.**
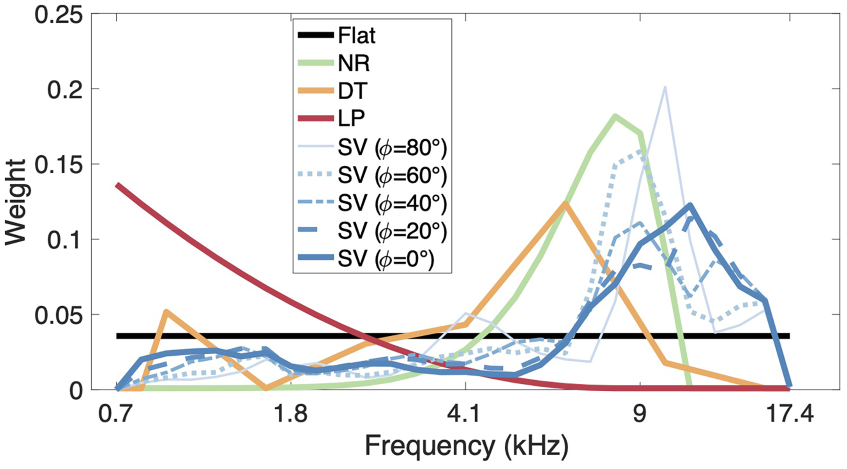
Spectral weighting schemes. NR: notch region; DT: discrimination task; LP: low-passed; SV: spatial variance calculated at group level for sagittal planes at various lateral angles $\phi_{k}$ , where $\phi_{k}$ = 0° represents the median plane.

We defined five model variants that differ by their spectral weighting scheme. Three weighting schemes were previously used or proposed by others and two schemes are proposed here. The weighting schemes are shown in Figure 2.

The first scheme previously proposed was taken from the original sagittal-plane localization model (Baumgartner et al., 2014), which assumes a flat spectral weighting. We refer to this scheme as the “Flat” scheme.

The second scheme was proposed by Zonooz et al. (2019). It emphasizes the frequency region where the main spectral notch changes with elevation and has a high weighting around 8 kHz. Thus, we refer to this scheme as the “NR” scheme.

The third scheme was derived from an elevation DT (Ahrens & Brimijoin, 2019), and it emphasizes frequencies around 6 kHz. Thus, we refer to this scheme as the “DT” scheme.

The fourth scheme was based on the potential effect of acoustic spatial variance (SV) on localization. We refer to this scheme as the “SV” scheme. It assumes that the frequency bands with larger SV in magnitude across polar angles provide more spatial information than those with a lower variance. To this end, we define the SV ν for each subject *i* and sagittal plane $\phi_{k}$ as:

$$\nu_{i}[\phi_{k},b]=(v_{i}^{L}[\phi_{k},b]\cdot \alpha +v_{i}^{R}[\phi_{k},b]\cdot (1-\alpha )+v_{i}^{L}[\phi_{-k},b]\cdot (1-\alpha )+v_{i}^{R}[\phi_{-k},b]\cdot \alpha )\cdot 0.5$$

where $v[\phi_{k},b]$ is the variance of ${\tilde{\xi }}_{r}[\phi_{k},\theta ,b]$ over the θ dimension, α is the binaural weight depending on the lateral angle (see section “Sagittal-plane localization model”). Note that $\nu_{i}[\phi_{k},b]=\nu_{i}[\phi_{-k},b]$ , thus, we were assuming symmetrical weights. The weights for each subject and sagittal plane were then computed as:

$$w_{i}[\phi_{k},b]=\frac{\nu_{i}[\phi_{k},b]}{\sum_{b=2}^{N_{b}}\;\nu_{i}[\phi_{k},b]}.$$

The fifth scheme is based on the observation that natural sounds typically exhibit a power spectrum gradually decaying with increasing frequency (Singh & Theunissen, 2003). Hence, one could potentially argue that listeners have learned to apply more weight to lower frequency regions in which, on average, the sound intensity is larger. Such a spectral weighting scheme was implemented as a decreasing exponential function, which is complementary to the other schemes, and we refer to it as the “Low Pass” (LP) scheme.

The schemes Flat, NR, DT, and LP were defined at the group level. For the scheme SV, subject-level information was available. We calculated the SV weights both at the subject and the group level in order to test whether the subject-level scheme helps to explain individual differences in localization behavior. While the subject-level scheme assumes that the contribution of each frequency band to sound-localization performance depends on the idiosyncratic localization cues of the listener, the group-level weights are the same for all subjects and were computed by averaging over the subject-level weights:

$$w[\phi_{k},b]=\frac{1}{N_{i}}\overset{N_{i}}{\underset{i=1}{\sum }}\;w_{i}[\phi_{k},b].$$

Figure 2 shows the SV scheme calculated at the group level for our subject pool in the median plane $\left(\phi_{k}=0\circ \right)$ and various sagittal planes ( $\phi_{k}$ = ±20°, ±40°, ±60°, ±80°). In the median plane, the group-level SV scheme shows a peak around 11 kHz. In more lateral sagittal planes, the peak occurs at lower frequencies, with a minimum of 9 kHz for $\phi_{k}$ = ±60°. Hence, with the increasing lateral angle, the SV scheme becomes similar to the NR scheme.

To decide whether the group- or subject-level weights should be used in the main analysis, a preselection process was performed in which we compared the predictions on these two types of weights. This comparison was done for median-plane localization data only.

### Parameter Estimation

Three model parameters were fitted: the degree of selectivity Γ , the sensitivity *S*, and the sensorimotor scatter ε . In Baumgartner et al. (2014), *S* was optimized for each subject, whereas Γ and ε were optimized at the group level to simplify the fitting procedure. In our study, by contrast, we relaxed this arbitrary constraint and allowed all three parameters to vary across individuals.

In Baumgartner et al. (2014), the parameter fitting was conducted by minimizing a cost function that combines the two-error metrics QE and PE. Yet those two metrics aggregate the listener's response behavior in a coarse manner. To make use of each listener's entire response pattern and fit all three parameters at the subject level, we here applied a fitting strategy based on likelihood maximization. The likelihood function was computed by using response probabilities provided by the model and the listener's directional responses recorded during the localization experiments.

The likelihood function L of the model is evaluated using the model's PMV and the actual responses. Thus, the likelihood for the j ^th^ actual response $\theta_{i,j}^{A}$ of subject *i* was computed as:

$$L(\theta_{i,j}^{A}|\Omega )=p_{\Omega }(\theta_{i,j}^{A}),$$

where Ω represents the model and $p_{\Omega }$ is the PMV. The optimized parameters were found as:

$$\{\Gamma ,S,\varepsilon {\}}_{i}^{\mathrm{opt}}=\underset{{\left\{\Gamma ,S,\varepsilon \right\}}_{i}}{\arg\;\max}\;\overset{N_{i,j}}{\underset{\;j=1}{\sum }}\;\ln\;(L(\theta_{i,j}^{A}|\Omega )).$$

Note that in contrast to Baumgartner et al. (2014), where QE and PE were considered within a large lateral range of −30° < ϕ < 30° around the median plane, our parameter fitting was done for a smaller lateral range of −10° < ϕ < 10°. This smaller lateral range was chosen so that sufficient remaining data were available to analyze the effect of the spectral weighting schemes outside the median plane.

The likelihood function optimization was performed using the Bayesian adaptive direct search algorithm (BADS; Acerbi & Ma, 2017). BADS alternates between a series of fast local optimization steps and a systematic slower exploration of a mesh grid defined on the parameter space. The model parameter ranges were defined as: 0.1 < Γ < 100; −20 < *S* < 20 and 3° < ε < 50°. These ranges were intended to represent the plausible parameter ranges while limiting the risk of getting stuck in local minima during the optimization procedure.

To check for differences in the optimized parameter values across conditions, a one-way analysis of variance test was conducted for each parameter (significance level: α=0.01 ). Post hoc comparisons were conducted using the Bonferroni correction for multiple comparisons.

### Model Variant Selection

Bayesian model selection was used to identify the model variant best predicting individual responses. Since the number of trials per listener differs across subjects (see Table 1), we used the Bayesian information criterion (BIC) as statistical metric. The BIC corrects the optimized likelihood depending on the number of samples and the number of fitted parameters, and, for each model variant and subject, the BIC resulted as:

$${\mathrm{BIC}}_{\Omega ,i}=\rho \cdot \ln\;N_{i,j}-2\cdot \overset{N_{i,j}}{\underset{\;j=1}{\sum }}\;\ln\;L(\theta_{i,j}^{A}|\Omega ),$$

where $N_{i,j}$ is the number of actual responses and ρ is the number of fitted parameters in the model ( ρ = 3 for all model variants).

In order to select which model variant best described measured data at the group level, we relied on a Bayesian selection method (Rigoux et al., 2014; Stephan et al., 2009). In contrast to the assumption of a single model best describing all subjects, this approach treats model variants as random effects that can vary across subjects by accounting for individual differences, offering a more flexible representation of the listeners’ pool. This method computes the protected exceedance probability (PXP) at the group level, that is, the corrected probability of preferring a model variant over the alternatives. The correction can be achieved with the computation of the Bayesian Omnibus Risk (BOR) quantifying the posterior probability that model frequencies are all equal. Thus, the per-listener PXP for each model variant was obtained by computing the posterior probability distribution *p* of the variants based on model evidence which informed which model best describes the individual response patterns:

$$p_{\Omega_{k},i}=\frac{{\mathrm{BIC}}_{\Omega_{k},i}}{\sum_{m=1}^{M}{\mathrm{BIC}}_{\Omega_{m},i}},$$

where k∈1,...,M is the index for the model being computed and M is the number of model variants. The group-level posterior distribution was obtained from these individual distributions while accounting for between-subject uncertainty following the procedure described in Rigoux et al. (2014).

The model selection was performed separately for the median plane only, that is, $\phi_{k}=0\circ$ , and sagittal planes outside the median plane, that is, for the lateral angles of $\phi_{k\;}$ = ±20°, ±40°, ±60°, ±80°. For each $\phi_{k}$ , the responses in the range $\phi_{k}$ −10° < ϕ < $\phi_{k}$ +10° were considered. For the model selection outside the median plane, the model parameters optimized for the median plane were used. Following the necessity to maintain each model's validity across our pool of subjects, we defined an exclusion criterion: A subject was excluded from the comparison if their parameter optimization did not converge within the defined boundaries for one of the compared model variants. Thus, subjects were not excluded because of low-likelihood fits, but only when their parameters converged outside the plausible bounds.

It was not clear whether the SV scheme should be defined at a group- or at a subject level. To address this question, a preselection was conducted to determine whether the SV scheme should be defined with the subject- or the group-level weights of the following analyses.

### Goodness of Fit

An analysis of the goodness of fit aimed at assessing the absolute performance of each model variant and at complementing the model variant selection by means of an intuitive metric of the benefit obtained by each of the model variants. For that, the coefficient of determination *R*^2^ was computed from log-likelihoods as in Nagelkerke (1991):

$$\begin{array}{rl} & R_{\Omega ,i}^{2}=1\\ & \;-\exp\left[-\frac{2}{N_{i,j}}\left\{\overset{N_{i,j}}{\underset{\;j=1}{\sum }}\;\ln(L(\theta_{i,j}^{A}|\Omega ))-\overset{N_{i,j}}{\underset{\;j=1}{\sum }}\;\ln(L(\theta_{i,j}^{A}|\Omega_{0}))\right\}\right], \end{array}$$

where $\Omega_{0}$ is the null model prescribing a uniform response distribution (i.e., simulating a participant making random localization responses). In order to achieve *R*^2^ = 1 for discrete models, *R*^2^ was corrected to ${\bar{R}}^{2}$ :

$${\bar{R}}_{\Omega ,i}^{2}=\frac{R_{\Omega ,i}^{2}}{1-\exp\;\{\;\frac{2}{N_{i,j}}\sum_{\;j=1}^{N_{i,j}}\;\ln(L(\theta_{i,j}^{A}|\Omega_{0}))\;\}}.$$

## Results

### Parameter Fits

**Figure 3.**
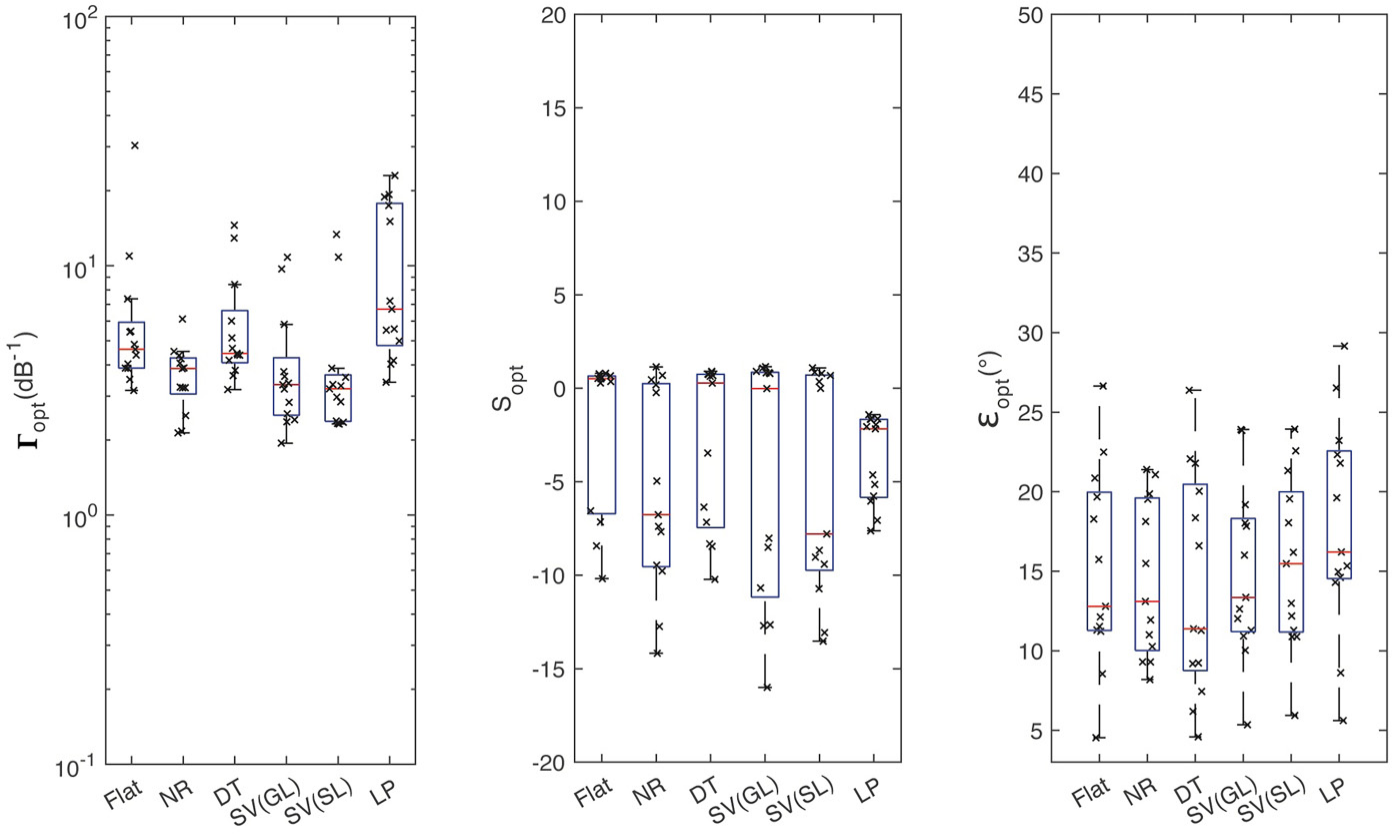
Statistics of the optimized model parameters for each of the model variants across subjects. The subjects with parameter fit not converging within predefined bounds (y-axis limits) were excluded. For SV, group-level (GL) and subject-level (SL) schemes were considered. The *S* parameter is dimensionless. The “x” symbols represent individual data.

The statistics of the parameters across subjects is shown in Figure 3. Note that only the subjects for which all parameters could be optimized (i.e., for all the weighting schemes) are included. For the Flat scheme, the optimization failed twice: ε for NH55 as well as Γ for NH58. For the NR scheme, the optimization of *S* failed for NH39. For the DT scheme, the optimization was successful for all the parameters. For the group-level SV scheme, the optimization failed in four cases: Γ for NH22, NH58, and NH57, as well as *S* for NH72. For the subject-level SV scheme, the optimization of Γ failed for NH14 and NH18 and ε failed for NH41 and NH62. For the LP scheme, the optimization of ε failed for NH55. After excluding these, predictions for 15 listeners remained available for further analyses. Although our exclusion criteria reduced the group sample size, the remaining still offered sufficient variability to effectively conduct our analysis. Parameter details are reported in the Supplementary Material.

Only the $\Gamma_{\mathrm{opt}}$ varied statistically significantly among conditions [*F*_(5,72)_ = 3.56, *p* < 0.01]. Post hoc comparisons showed that the distribution of $\Gamma_{\mathrm{opt}}$ for the LP condition was different from that for the NR, group-level SV, and subject-level SV conditions. No other pairwise differences were found to be significant.

### Effect of the Spectral Weighting Schemes and Parameters on the PMVs

**Figure 4.**
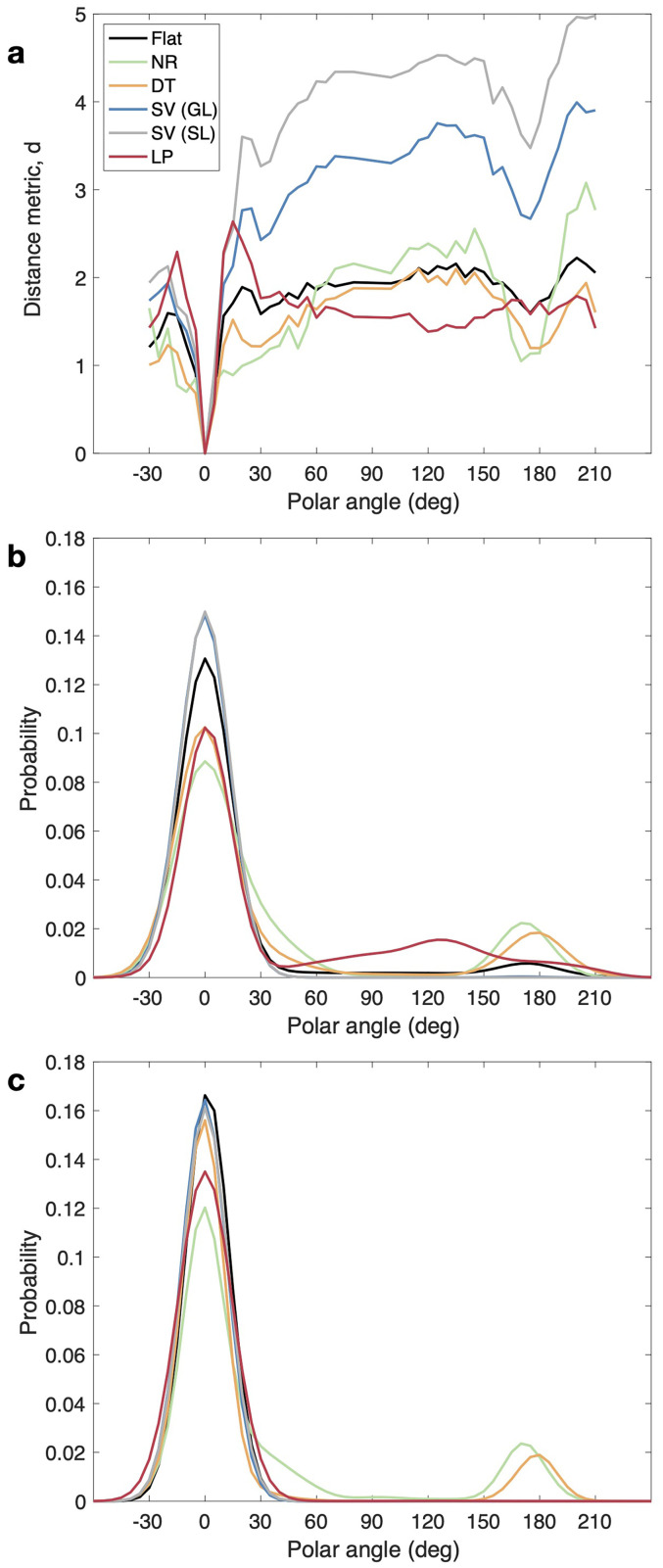
Examples of the effect of the spectral weighting schemes on the probability mass vectors (PMVs). Panel (a) shows the model's internal distance values for a target located at θ = 0° in the median plane for the example listener NH12 (left ear), and all the studied spectral weighting schemes (bin size = 5°): Flat, NR: notch region, DT: discrimination task, SV (GL): spatial variance (group level), SV (SL): spatial variance (subject level), and LP: low-passed. Panel (b) shows the PMVs for fixed parameters (median parameters obtained for the Flat weighting scheme). Panel (c) shows the resulting PMVs for optimized parameters (see Table 2 in Supplemental Material for the specific parameter values).

The effect of the spectral weighting schemes on the PMVs for the example listener NH12 is shown in Figure 4. Panel (a) shows the distance metric for each model variant and the target located at the frontal direction (i.e., θ = 0°). The highest similarity was consistently found at the target direction, as expected for the present combinations of broadband flat-spectrum stimuli and broadband spectral weighting schemes. However, for the other polar angles, there were clear differences among the model variants. Note that the model does not just select the minimum of this distance distribution to generate a response prediction, on the contrary, it transforms the whole distribution into a response PMV, thus, remains sensitive to the different spectral weighting schemes. Panel (b) shows the PMVs calculated with parameters (medians over the listeners) obtained from the optimization of the Flat model variant. Panel (c) shows the PMVs calculated with the parameters optimized individually for each model variant (see Table 2 in Supplemental Material). The differences between the two panels underline the importance of the individual parameter optimization for each of the weighting schemes.

### Preselection of the SV Scheme: Group vs. Subject Level

**Figure 5.**
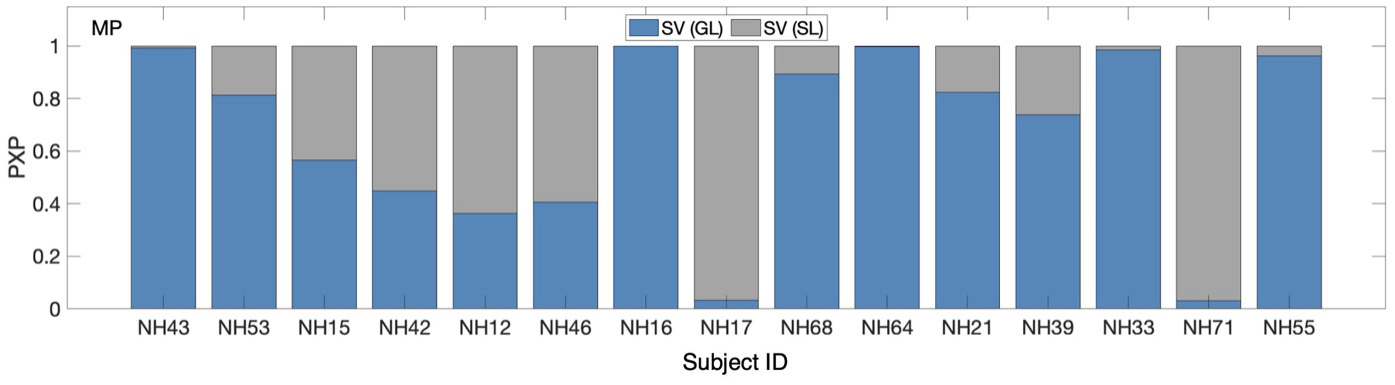
Protected exceedance probabilities (PXPs) of a model variant being best at describing the actual responses in the localization task. Results of the pre-selection of two model variants with the SV scheme are shown: that one with weights calculated at the group level (GL) and that one at the subject level (SL).

The preselection analysis resulted in a PXP = 0.66 in favor of the group-level SV, that is, the probability of group-level SV being better at describing the data than the subject-level SV was 0.66. However, the probability of selecting the wrong model, BOR, was 0.64, which was considered too high to conclude that one model variant is better than the other one. Figure 5 shows the results of this inconclusive comparison: the responses of some listeners were better explained by individual weights and of others by group-level weights.

Despite this inconsistency, in order to enable a fairer comparison with the weighting schemes available at the group level only, the group-level SV scheme was selected for further analyses. This also enabled us to reinclude all the listeners for which the parameters have been optimized within the plausible boundaries for the group-level SV scheme.

### Model Selection

**Figure 6.**
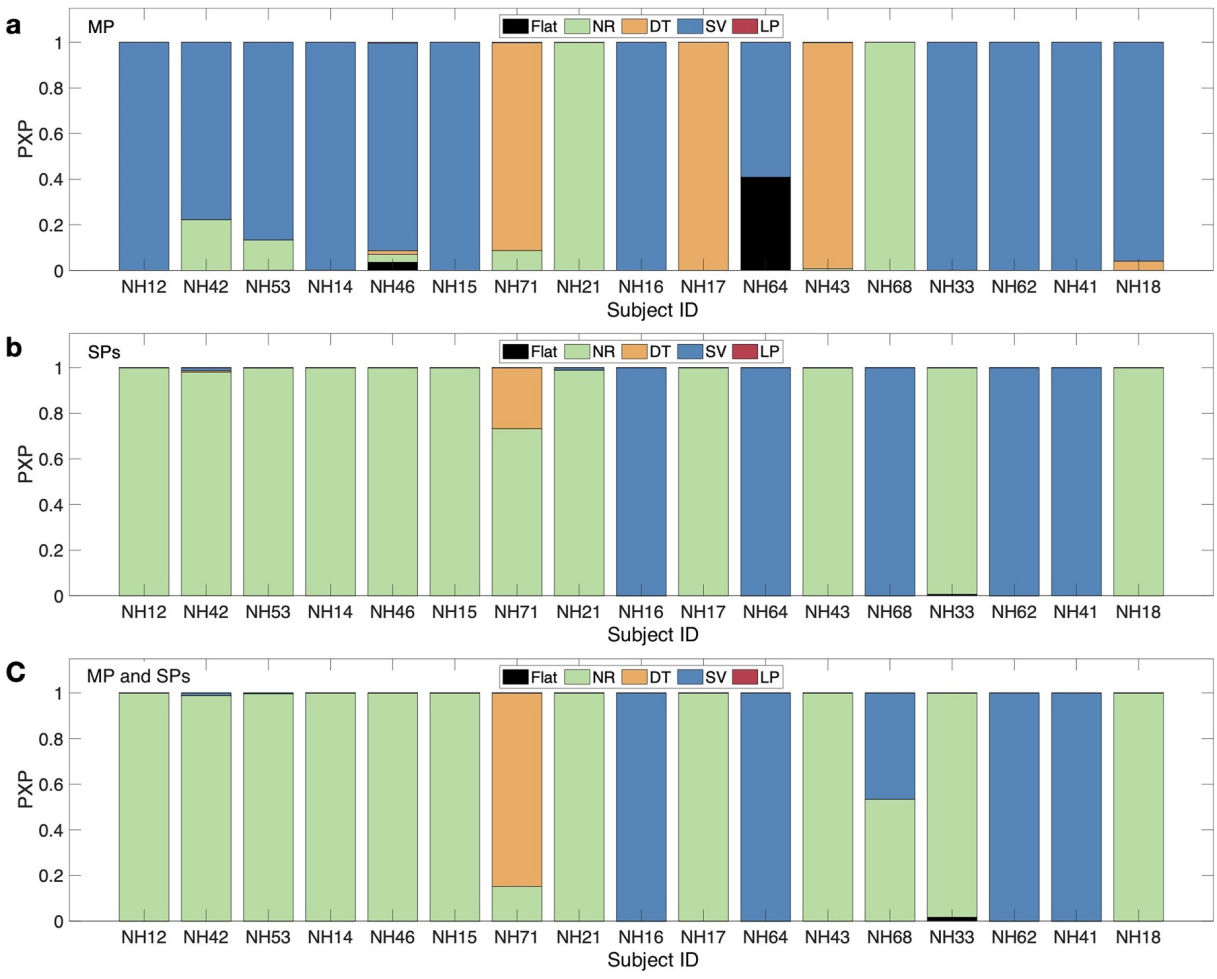
Protected exceedance probabilities (PXPs) of a model variant being best at describing the actual responses in the localization task. Results of the comparison of five model variants: Flat, NR: notch region, DT: discrimination task, SV: spatial variance, and LP: low-passed. Panel (a) shows the results of analyzing the median plane; panel (b) shows the results of analyzing the lateral sagittal planes ( $\phi_{k}$ = ±20°, ±40°, ±60°, ±80°).

The PXPs for the model variants applied on the median-plane data only, which is the region for which the parameter values were optimized, are shown in Figure 6(a). The SV scheme was the most often preferred scheme (12 out of 17 subjects) as supported by the high group-level PXP of 0.96 and low BOR of 0.01. For two subjects, NR was the preferred scheme; for three subjects, DT was the preferred scheme.

The PXPs for the various model variants in the lateral sagittal planes, that is, $\phi_{k}$ = ±20°, ±40°, ±60°, ±80°, are shown in Figure 6(b). Note that the predictions for the sagittal planes were not used for the parameter optimization. The model selection showed a clear preference for the NR scheme for 12 out of 17 subjects, as supported by the high group-level PXP of 0.94 and a low BOR (smaller than 0.01). For five subjects, SV was the preferred scheme.

For a joint analysis across all lateral angles, Figure 6(c) shows the PXPs obtained from predictions done for targets located on the median and lateral sagittal planes. This combined model selection showed a clear preference for the NR scheme for 12 out of 17 subjects, with a group-level PXP of 0.96 and a low BOR (smaller than 0.01). For four subjects, the SV was the preferred scheme. For one subject, the DT was the preferred scheme.

### Goodness of Fit

**Figure 7.**
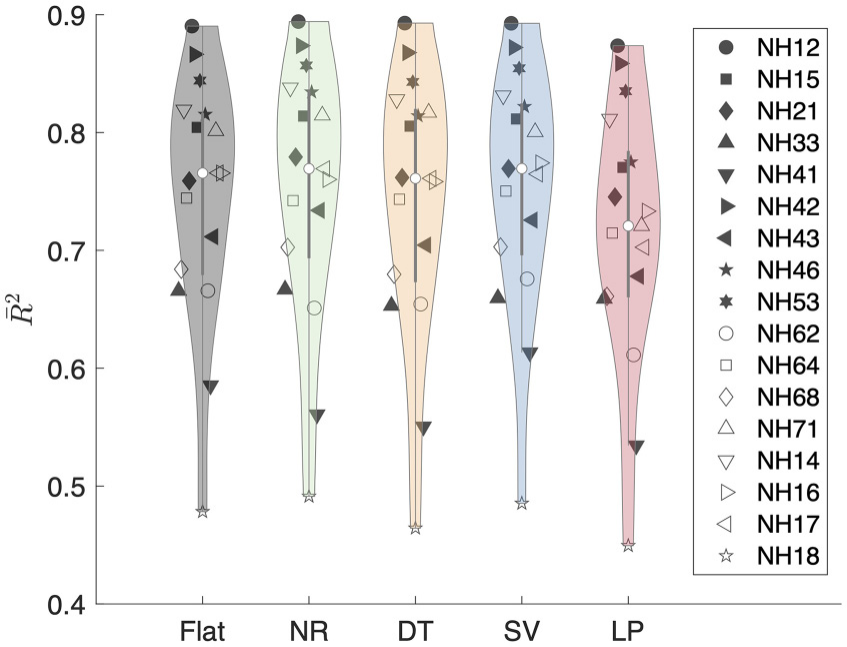
Nagelkerke's corrected ${\bar{R}}^{2}$ values for each of the model variants: flat, NR: notch region, DT: discrimination task, SV: spatial variance and LP: low-passed. Panel (a) shows the results of analyzing the median plane; panel (b) shows the results of analyzing the lateral sagittal planes ( $\phi_{k}$ = ±20°, ±40°, ±60°, ±80°).

The ${\bar{R}}^{2}$ are shown in Figure 7 for median-plane and sagittal-plane targets combined. The medians over all listeners varied only a little, being smallest for ${\bar{R}}_{\mathrm{LP}}^{2}$ of 0.72 and largest for ${\bar{R}}_{\mathrm{SV}}^{2}$ and ${\bar{R}}_{\mathrm{NR}}^{2}$ both being at 0.77. This indicates that all model variants were able to account for a good amount of the variability present in the collected responses.

However, ${\bar{R}}^{2}$ showed a large intersubject variability, ranging from ${\bar{R}}_{\mathrm{NR}}^{2}$ of 0.49 for NH18 to ${\bar{R}}_{\mathrm{NR}}^{2}$ of 0.89 for NH12 (with NR being the preferred scheme for both subjects). Within each listener, the variability over model variants was rather low: for a single listener, the largest ${\bar{R}}^{2}\;$ was 0.1, indicating that the subject had a larger impact than the scheme.

Interestingly, there was no clear pattern of interaction between ${\bar{R}}^{2}$ and the best model. This is demonstrated in Figure 6(c), in which the subject's position on the abscissa was sorted by ${\bar{R}}^{2}$ . Altogether, these results indicate that the preferred spectral weighting scheme does not depend on the model's goodness of fit and that differences in schemes can only explain a small portion of interindividual differences.

### Actual vs. Predicted Localization Patterns

**Figure 8.**
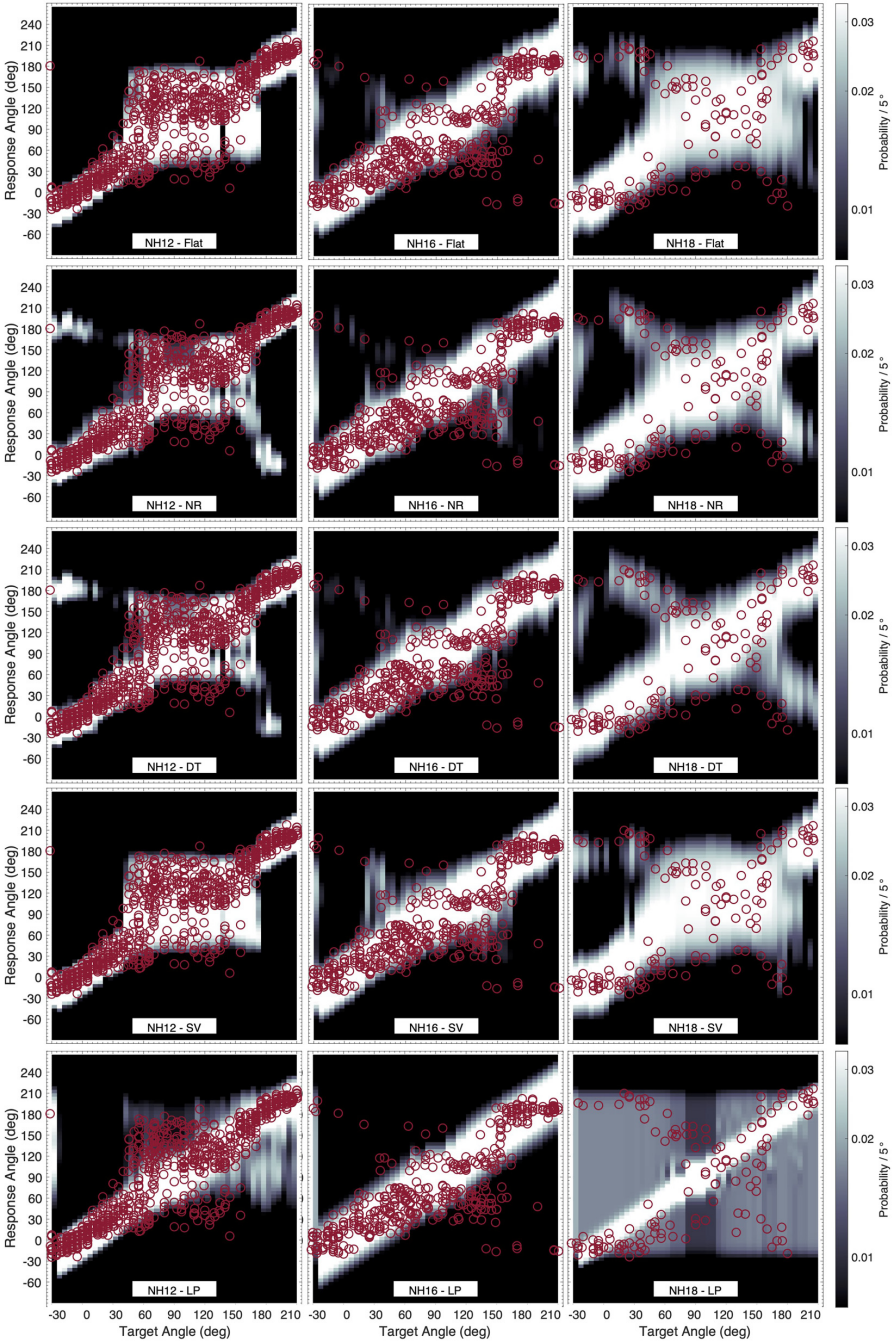
Examples of predicted probability mass vectors (PMVs; encoded by brightness) obtained from the five model variants (in rows) for the responses of three listeners (in columns: NH21, NH41, and NH18). Actual responses are shown as red open circles.

Figure 8 shows the effect of the weighting schemes on the model PMVs in the median plane for the listeners NH12 (highest ${\bar{R}}^{2}$ , *N* = 721 trials), NH16 (median ${\bar{R}}^{2}$ , *N* = 414), and NH18 (lowest ${\bar{R}}^{2}$ , *N* = 136). For NH12, the PMVs obtained with the Flat and SV schemes seem to capture the response pattern best: higher localization precision in the ranges −30° < θ < 45° and 180° < θ < 210°, and lower precision in the range 45° < θ < 180°. The PMVs obtained with the NR and DT weighting schemes are similar, predicting a front-back confusion pattern that is not present in the listener's responses. The PMVs obtained with the LP weighting scheme show a pattern with particularly low precision for rear targets, contradicting the actual response patterns.

For NH16, the different spectral weighting schemes produced largely similar PMVs, with precision remaining relatively constant along with the entire median plane. However, particularly for upper rear target angles, the PMVs obtained with the SV scheme seem to best (though still only partially) capture the consistent bias toward upper frontal responses.

For NH18, the response pattern shows a high front-back confusion rate in the ranges −30° < θ < 90° and 150° < θ < 210°, and low precision in the range 90° < θ < 150°. This pattern seems to be captured by the PMVs obtained with the Flat, NR, DT, and SV schemes. The PMVs obtained with the LP scheme do not capture this trend. Instead, it predicts high uncertainty for the entire polar range. The response pattern also shows a bias toward the horizontal plane in the range −30° < θ < 45°, which is not captured by any of the model variants and instead approximated as a general lack of precision.

## Discussion

A model-based comparison of spectral weighting schemes showed a consistent yet small benefit in emphasizing the contribution of spectral cues at higher frequencies for sagittal-plane sound localization of wideband, flat-spectrum targets. The best predictions were obtained when the contribution of the spectral cues was emphasized at higher frequencies. Emphasizing the contribution of the main NR (around 8 kHz) provides the best predictions, with those emphasizing the largest SV (around 11 kHz) being the second-best choice.

### Weighting Schemes

Our results show that weighting schemes emphasizing the contribution of spectral cues at higher frequencies outperform the flat spectral weighting used in Baumgartner et al. (2014). These results suggest that listeners have an increased sensitivity to spectral localization cues at higher frequencies, being in accordance with results from Zonooz et al. (2019) and Van Opstal et al. (2017). Thus, special attention should be put on accurately reproducing the high-frequency region of spectral-shape cues that listeners seem to upweight in their spatial perceptual evaluation. It is worth mentioning that only young adults with normal-hearing participated in this study, and our results might not generalize for other population groups such as older listeners or listeners suffering from hearing loss in high frequencies.

The SV scheme yielded best predictions for 5 out of 17 subjects only. For the majority, namely 12 out of 17 subjects, best predictions were achieved by the NR scheme, that is, by emphasizing the contribution of the main notch. The corresponding frequency range covers the region of a monotonic increase in the main notch center frequency with the elevation angle. It thus appears to be particularly useful for estimating source elevation (Van Opstal et al., 2017; Zonooz et al., 2019). In the particular case of the median plane (region where the model parameters were optimized), the most preferred weighting was the SV scheme. That SV scheme has largest weights around 11 kHz, which falls under a frequency region that was found to provide useful information for elevation perception in Van Opstal et al. (2017).

The increase in sensitivity of specific spectral regions can be interpreted as optimization of perceptual inference in response to specific acoustic conditions (Keating et al., 2013). Such optimization mechanisms have been shown for the binaural weighting of spectral cues, where the contribution of each ear depends on the interaural cues (Li et al., 2020; Macpherson & Sabin, 2007; Morimoto, 2001). Similarly, a reweighting of localization cues seems to be required to maintain a stable perception of the auditory space (Kumpik et al., 2010). When listeners are exposed to abnormal conditions that persist over time, such as unilateral earplugging (Kumpik et al., 2010) or deliberate altered localization cues (Klingel & Laback, 2022), their localization performance improves by adapting to using the most reliably available cues. Therefore, such adaptation mechanism may also exist for the integration of spectral cues by relying on the most informative frequency regions, for example, the main NR (Zonooz et al., 2019) or based on the SV. However, if the optimization of perceptual inference was responsible for the spectral weights, one could expect that the subject-level SV scheme should have outperformed the group-level SV scheme. Unfortunately, this is not clear from our results. It is possible that the individual schemes are susceptible to containing outliers (i.e., atypical values for specific listeners found within specific frequency bands), and pooling across subjects helped to average those out.

The analysis of the goodness of fit showed similar performance across model variants, indicating that the benefit of selecting among spectral weighting schemes is rather subtle. The small differences in ${\bar{R}}^{2}$ are likely due to the fact that all model variants shared most stages of the original model implementation and were optimized to the same response distributions using the same approach.

### Interindividual Differences

Our results show interindividual differences both in the goodness of fit and the model selection results. While all tested model variants seem to capture reasonably well the response patterns for some listeners, they showed a poor fit for others. These differences could be related to the listeners' localization abilities and/or their strategies to perform the task. As shown in Majdak et al. (2014), nonacoustic factors, that is, factors that are related to perceptual abilities and not to the HRTF itself, seem to play an important role. Certain errors are not properly captured by the model. This concerns particular biases in the response patterns (e.g., see Figure 8, in which the response pattern for NH18 presents a bias toward the horizontal plane that is not captured by the PMVs). Further steps could account for biases in the model, for example, as in Barumerli et al. (2023), but our conclusions regarding weighting schemes seem not to be compromised by this inability to model such responses.

Our model selection results show an improvement in the predictions when the high-frequency region is emphasized, but there are inter-individual differences in which model best describes a listener's response pattern. The origin of these differences is unclear. They may be related to acoustic factors, that is, features that have an impact on the HRTFs, such as pinnae size (Best et al., 2005; Zhang & Hartmann, 2010). However, these could also have a non-acoustic origin, for example, some listeners may focus more on the NR, while others may better exploit the available SV.

The result of the subject- vs. group-level SV was inconclusive. We hypothesized that listeners may learn to exploit frequency bands with higher spectral variance in their HRTF. While adaptation to HRTFs is a long-life process (Mendonça, 2014), it remains unclear whether listeners learn to take advantage of the specific frequency bands at which their HRTFs present larger spectral variance for localization tasks.

### Limitations and Future Directions

We were not able to fit parameters for the data in 12 (out of 138) cases, that is, combinations of subjects and model variants. Two of those cases were related to a missing convergence for the parameter *S*, six are due to Γ , and four due to ε . The two parameters *S* and Γ define a single mapping function and thus can affect each other in the sense that a small Γ may reduce the effect of *S* (Baumgartner et al., 2014). Consequently, for the two cases with non-converging *S*, the parameter Γ converged at the minimum. In the future, optimization strategies better accounting for the dependency of these two parameters could help in fitting parameters for such variants. The parameter ε did not converge in two occasions for the same subject (NH55). For this subject, a very low number of actual responses was available, which, combined with the individual scatter of responses, might have led to the non-convergence.

In this study, the model parameters were considered constant across all sagittal planes. This might be a simplification because the model parameters may vary along with some dimensions. For example, it might be that the sensorimotor scatter changes with the lateral angle or even differ between left and right. In future modeling approaches, it might be interesting to investigate such dependencies, their effect on the predicted localization performance, and their contribution to the spectral weighting schemes.

We focused on wideband flat-spectrum stimuli, which is a specific case for which localization performance is usually accurate. Under such conditions, listeners know the frequency content of the stimulus a priori, which may influence the cues they exploit in the task. In everyday listening situations, spectral-weighting schemes may change depending on the task and the spectral content of the target. The latter may be a crucial aspect because the frequency bands with a low signal-to-noise ratio may be ignored by the auditory system in the process of sound-source localization. Previous studies varied systematically the frequency content of the stimuli to find which frequency regions are necessary for accurate localization or how the performance drops when the bandwidth is limited (Hebrank & Wright, 1974; Langendijk & Bronkhorst, 2002; Zhang & Hartmann, 2010). While such studies are of great value to understand which frequency regions need to be delivered to the listener, conclusions about the contribution of each specific frequency region within a broadband stimulus remain quite speculative. This and our methodology could be combined in the future to understand how the contribution of each specific frequency region depends on the frequency content of the stimulus.

Further studies should assess the generalizability of our findings to stimuli with limited bandwidth and spectral variations, for example, by simultaneously presenting two (or more) competing sounds with limited non-overlapping bandwidths and analyzing which frequency regions—emphasized by each spectral weighting scheme—dominate the localization responses. This would complement our data by extending the scope of the study from Ahrens & Brimijoin (2019). Moreover, the spectral content of the stimuli could be systematically varied over trials to understand to which extent the contribution of each frequency range depends on prior knowledge.

Taken together, our results suggest that an increase in the perceptual weight of frequency regions with systematic changes in spectral cues is relevant for accurate modeling of sagittal-plane sound localization. Thus, further development in HRTF individualization procedures need to be particularly accurate in this frequency region.

## Supplemental Material

sj-docx-1-tia-10.1177_23312165251317027 - Supplemental material for Spectral Weighting of Monaural Cues for Auditory Localization in Sagittal Planes Supplemental material, sj-docx-1-tia-10.1177_23312165251317027 for Spectral Weighting of Monaural Cues for Auditory Localization in Sagittal Planes by Pedro Lladó, Piotr Majdak, Roberto Barumerli and Robert Baumgartner in Trends in Hearing
