# Supplementary material for: Spectral Weighting of Monaural Cues for Auditory Localization in Sagittal Planes
Source: Trends Hear. 2025 Mar 18;29:23312165251317027. doi: 10.1177/23312165251317027 (PMC11920987; doi:10.1177/23312165251317027)
Supplement: sj-docx-1-tia-10.1177_23312165251317027 - Supplemental material for Spectral Weighting of Monaural Cues for Auditory Localization in Sagittal Planes [file sj-docx-1-tia-10.1177_23312165251317027.docx]

**Table 2 (Supplementary material)**. Optimized parameters (, degree of selectivity; , sensitivity; and , sensorimotor scatter) for each subject and model variant using the Bayesian adaptive direct search algorithm (Acerbi & Ma, 2017). The N column shows the number of responses in the median plane ±10°. The * symbol refers to parameters which optimization did not converge within the defined parameter range. The median model parameters (last row) were computed after excluding the data from subjects yielding non-converging parameter fits. The listeners are sorted by QE for the median plane localization data.

|  |  | Flat | | | NR | | | DT | | | SV (GL) | | | SV (SL) | | | LP | | |
| --- | --- | --- | --- | --- | --- | --- | --- | --- | --- | --- | --- | --- | --- | --- | --- | --- | --- | --- | --- |
|  | N |  |  |  |  |  |  |  |  |  |  |  |  |  |  |  |  |  |  |
| NH43 | 48 | 4.84 | 0.28 | 12.13 | 3.87 | 0.18 | 11.01 | 14.53 | 0.76 | 11.29 | 2.83 | -10.68 | 13.36 | 2.84 | -10.72 | 15.48 | 4.99 | -6.04 | 14.31 |
| NH53 | 79 | 7.36 | 0.58 | 15.74 | 3.89 | -7.39 | 15.49 | 4.17 | -3.47 | 16.6 | 3.38 | -8.5 | 16.01 | 3.3 | -8.67 | 16.19 | 17.44 | -1.66 | 21.8 |
| NH58 | 74 | 100.0* | 0.81 | 12.78 | 10.86 | 0.48 | 15.71 | 12.14 | 0.58 | 15.48 | 100.0* | 0.89 | 15.22 | 9.15 | 0.87 | 13.16 | 7.67 | -1.92 | 17.81 |
| NH15 | 383 | 10.94 | 0.66 | 20.86 | 6.11 | 0.45 | 19.52 | 12.91 | 0.64 | 21.79 | 5.82 | 0.83 | 17.84 | 13.35 | 0.8 | 21.33 | 6.7 | -2.17 | 26.52 |
| NH42 | 278 | 4.62 | -6.56 | 18.28 | 4.37 | -4.96 | 18.13 | 4.39 | -6.36 | 18.36 | 3.76 | -8.02 | 18.03 | 3.88 | -7.79 | 18.05 | 18.88 | -1.64 | 19.62 |
| NH12 | 721 | 30.37 | 0.8 | 11.3 | 3.24 | -0.23 | 9.3 | 8.4 | 0.68 | 9.24 | 10.81 | 0.9 | 11.29 | 10.83 | 0.68 | 12.19 | 7.22 | -1.95 | 14.61 |
| NH46 | 50 | 3.16 | -10.18 | 4.54 | 3.24 | -9.77 | 9.3 | 3.19 | -10.22 | 4.59 | 2.55 | -12.65 | 5.35 | 3.57 | -9.03 | 5.94 | 23.0 | -1.41 | 5.61 |
| NH16 | 414 | 5.42 | 0.36 | 19.67 | 4.23 | -6.77 | 19.86 | 6.01 | 0.28 | 20.03 | 3.33 | -0.02 | 19.18 | 3.21 | 0 | 19.55 | 15.05 | -2.05 | 23.22 |
| NH14 | 44 | 15.03 | 0.83 | 12.84 | 2.91 | -4.64 | 11.09 | 3.63 | -3.75 | 13.75 | 14.98 | 1.06 | 12.15 | 100.0* | 1.06 | 12.59 | 5.57 | -2.32 | 16.12 |
| NH17 | 150 | 3.88 | 0.53 | 8.56 | 2.13 | -14.18 | 11.94 | 4.66 | 0.74 | 9.19 | 1.94 | -16.0 | 10.03 | 2.33 | -13.53 | 10.89 | 4.17 | -7.62 | 8.61 |
| NH57 | 42 | 17.96 | 1.01 | 10.31 | 3.68 | -8.01 | 14.2 | 3.7 | -7.9 | 13.66 | 100.0* | 1.54 | 8.59 | 2.54 | -0.45 | 12.95 | 5.45 | -2.78 | 13.14 |
| NH68 | 98 | 3.5 | -8.43 | 12.79 | 3.26 | -9.46 | 10.26 | 3.62 | -8.32 | 11.38 | 2.41 | -12.7 | 10.93 | 2.36 | -13.07 | 10.88 | 5.52 | -4.63 | 14.96 |
| NH64 | 141 | 5.46 | 0.77 | 11.53 | 4.53 | 1.14 | 13.11 | 5.14 | 0.87 | 7.45 | 3.6 | 1.15 | 12.63 | 2.97 | 1.09 | 12.98 | 4.03 | -7.06 | 15.34 |
| NH62 | 101 | 2.38 | -13.16 | 13.92 | 2.95 | -10.92 | 13.4 | 2.6 | -11.98 | 14.12 | 1.64 | -19.15 | 11.87 | 1.72 | -18.39 | 12.56 | 9.57 | -3.36 | 20.9 |
| NH22 | 187 | 8.37 | 0.76 | 15.41 | 2.73 | -5.98 | 14.91 | 3.57 | -0.19 | 14.14 | 100.0* | 1.14 | 19.52 | 2.66 | -0.33 | 14.62 | 6.65 | -4.79 | 13.28 |
| NH21 | 141 | 4.38 | -7.15 | 22.49 | 4.06 | -7.67 | 21.08 | 4.38 | -7.17 | 22.06 | 9.71 | 1.08 | 23.9 | 3.34 | -9.41 | 22.57 | 19.3 | -1.65 | 22.34 |
| NH72 | 117 | 2.45 | -12.82 | 9.46 | 2.37 | -13.35 | 15.37 | 2.42 | -12.85 | 10.21 | 1.59 | -20.0* | 11.84 | 2.97 | -10.57 | 22.92 | 7.65 | -4.26 | 11.55 |
| NH39 | 231 | 2.12 | -10.24 | 16.62 | 1.6 | -20.0* | 15.34 | 2.58 | -12.63 | 13.64 | 1.17 | -15.15 | 14.12 | 1.15 | -14.39 | 14.42 | 5.94 | -5.46 | 17.69 |
| NH33 | 149 | 3.88 | 0.51 | 26.64 | 2.51 | -12.74 | 21.39 | 3.8 | -8.45 | 26.37 | 3.2 | 0.82 | 23.85 | 2.38 | 0.35 | 23.93 | 5.57 | -5.77 | 29.16 |
| NH71 | 47 | 4.03 | 0.65 | 11.22 | 2.17 | 0.69 | 8.19 | 4.45 | 0.9 | 6.19 | 2.37 | 0.79 | 12.02 | 2.32 | 0.85 | 11.29 | 3.41 | -5.15 | 16.21 |
| NH55 | 43 | 5.48 | 1.03 | 3.0* | 3.3 | -9.66 | 22.74 | 3.86 | 0.56 | 18.83 | 4.59 | 1.33 | 18.74 | 1.57 | -20.0* | 19.18 | 3.52 | 0.43 | 3.0* |
| NH18 | 136 | 22.42 | 1.15 | 22.7 | 3.9 | 0.94 | 25.38 | 4.85 | 0.76 | 24.85 | 17.14 | 1.59 | 22.05 | 100.0* | 1.71 | 21.02 | 8.91 | -3.78 | 10.24 |
| NH41 | 139 | 1.85 | -17.46 | 7.21 | 1.83 | 0.37 | 6.35 | 4.7 | 0.92 | 6.05 | 1.07 | -11.53 | 6.46 | 0.88 | -20.0* | 6.38 | 4.27 | -7.9 | 9.74 |
| Median | 136 | 4.62 | 0.51 | 12.79 | 3.87 | -6.77 | 13.11 | 4.45 | 0.28 | 11.38 | 3.33 | -0.02 | 13.36 | 3.21 | -7.79 | 15.48 | 6.70 | -2.17 | 16.21 |
